# Supplementary material for: Aldh2 Attenuates Stem Cell Factor/Kit-Dependent Signaling and Activation in Mast Cells
Source: Int J Mol Sci. 2019 Dec 10;20(24):6216. doi: 10.3390/ijms20246216 (PMC6940998; doi:10.3390/ijms20246216)
Supplement: Supplementary file 1 [file ijms-20-06216-s001.pdf]

Supplementary

Aldh2 Attenuates Stem Cell Factor/Kit Dependent Signaling

Do-Kyun Kim [1,†,*], Young-Eun Cho [2,‡], Byoung-Joon Song [2], Toshihiro Kawamoto [3], Dean D. Metcalfe [1] and Ana Olivera [1,*]

1. Mast Cell Biology Section, Laboratory of Allergic Diseases, National Institute of Allergy and Infectious Diseases (NIAID), National Institutes of Health (NIH), Bethesda, MD 20892, USA; dmetcalfe@niaid.nih.gov (D.D.M.)
2. Section of Molecular Pharmacology and Toxicology, Laboratory of Membrane Biochemistry and Biophysics, National Institute on Alcohol Abuse and Alcoholism (NIAAA), NIH, Bethesda, MD 20892, USA; yecho@andong.ac.kr (Y.-E.C.); bj.song@nih.gov (B.-J.S.)
3. Occupational Health Research and Development Center, Japan Industrial Safety and Health Association, Tokyo 108-0014, Japan; t-kawamoto@jisha.or.jp (T.K.)

† Current address: Center for Biomolecular & Cellular Structure, Institute for Basic Science (IBS), Daejeon 34126, South Korea.

‡ Current address: Department of Food and Nutrition, Andong National University, Andong, Kyungpook 760-749, South Korea.

* Correspondence: dkkim0912@ibs.re.kr (D.-K.K); Ana.Olivera@nih.gov (A.O.)

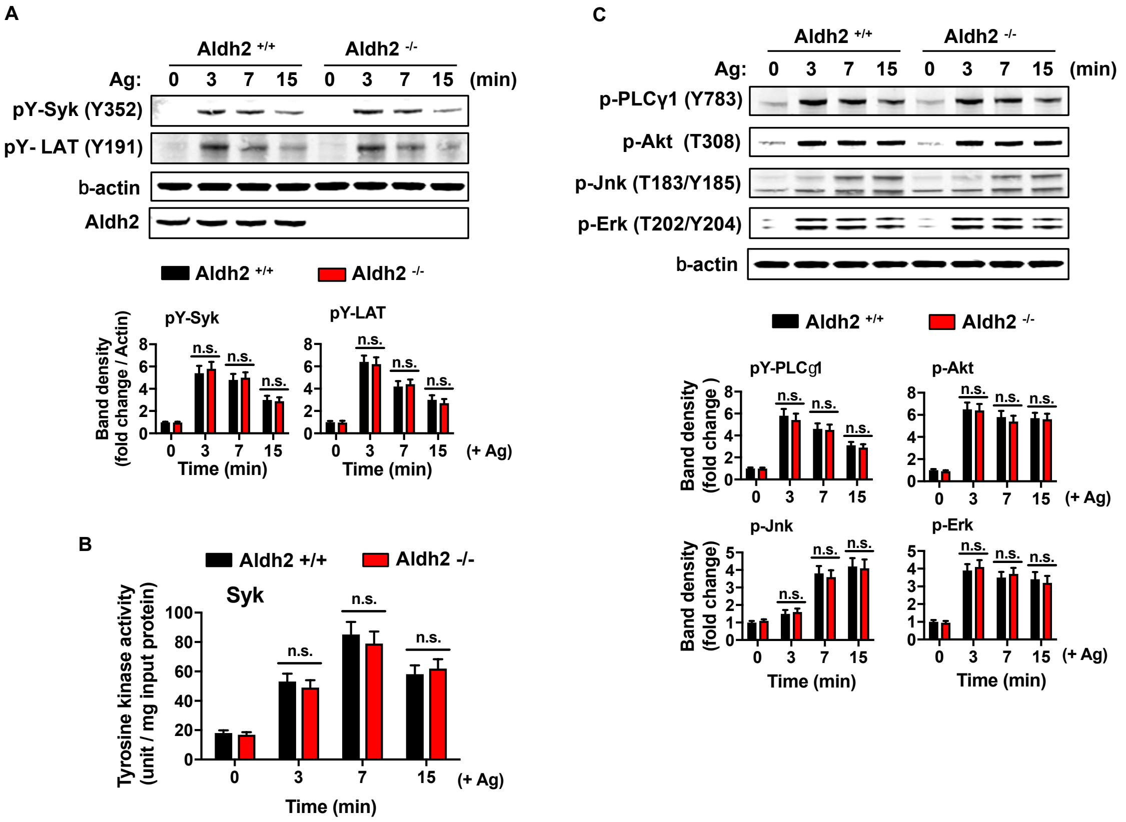

Figure S1. Aldh2 deficiency does not alter FcεRI-mediated signaling in BMMC. (**a**) Changes in the phosphorylation of Syk and LAT at 0, 3, 7 and 15 min after activation with IgE/Ag of Aldh2$^{+/+}$ or Aldh2$^{-/-}$ BMMCs, as indicated. BMMCs were sensitized with anti-DNP IgE (100 ng/mL) overnight, then washed and challenged with 100 ng/mL of Ag (DNP-HSA). Lysates were obtained and phosphorylation of Syk and LAT determined by Western blotting. The histograms below show the

average fold changes in band intensities after normalization using βactin as a loading control. (**b**) Changes in Syk tyrosine kinase phosphorylation in immunoprecipitates of Aldh2$^{+/+}$ or Aldh2$^{-/-}$ BMMCs after stimulation with IgE/Ag for various times, as indicated. (**c**) Changes in the phosphorylation of PLCγ1, Akt, Jnk and Erk at 0, 3, 7 and 15 min after activation with IgE/Ag of Aldh2$^{+/+}$ or Aldh2$^{-/-}$ BMMCs. Lysates were obtained and phosphorylation of Syk and LAT determined by Western blotting. The histograms below show the average fold changes in band intensities after normalization using β-actin as a loading control. Data are the mean ± SEM of 3 independent cultures. N.s., not significant.
